# Phenotyping a Dynamic Trait: Leaf Growth of Perennial Ryegrass Under Water Limiting Conditions

**DOI:** 10.3389/fpls.2019.00344

**Published:** 2019-03-22

**Authors:** Steven Yates, Kristina Jaškūnė, Frank Liebisch, Sebastian Nagelmüller, Norbert Kirchgessner, Roland Kölliker, Achim Walter, Gintaras Brazauskas, Bruno Studer

**Affiliations:** ^1^Molecular Plant Breeding, Department of Environmental Systems Science, Institute of Agricultural Sciences, ETH Zurich, Zurich, Switzerland; ^2^Laboratory of Genetics and Physiology, Institute of Agriculture, Lithuanian Research Centre for Agriculture and Forestry, Akademija, Lithuania; ^3^Crop Science, Institute of Agricultural Sciences, Department of Environmental Systems Science, ETH Zurich, Zurich, Switzerland

**Keywords:** drought tolerance, leaf elongation rate (LER), monocots, leaf growth, perennial ryegrass (*Lolium perenne* L.), phenotyping, tri-phase function, water deficit

## Abstract

Water limitation is one of the major factors reducing crop productivity worldwide. In order to develop efficient breeding strategies to improve drought tolerance, accurate methods to identify when a plant reduces growth as a consequence of water deficit have yet to be established. In perennial ryegrass (*Lolium perenne* L.), an important forage grass of the Poaceae family, leaf elongation is a key factor determining plant growth and hence forage yield. Although leaf elongation has been shown to be temperature-dependent under non-stress conditions, the impact of water limitation on leaf elongation in perennial ryegrass is poorly understood. We describe a method for quantifying tolerance to water deficit based on leaf elongation in relation to temperature and soil moisture in perennial ryegrass. With decreasing soil moisture, three growth response phases were identified: first, a “normal” phase where growth is mainly determined by temperature, second a “slow” phase where leaf elongation decreases proportionally to soil water potential and third an “arrest” phase where leaf growth terminates. A custom R function was able to quantify the points which demarcate these phases and can be used to describe the response of plants to water deficit. Applied to different perennial ryegrass genotypes, this function revealed significant genotypic variation in the response of leaf growth to temperature and soil moisture. Dynamic phenotyping of leaf elongation can be used as a tool to accurately quantify tolerance to water deficit in perennial ryegrass and to improve this trait by breeding. Moreover, the tools presented here are applicable to study the plant response to other stresses in species with linear, graminoid leaf morphology.

## Introduction

Water limitation is a major factor reducing the yield of crop species worldwide (Lobell et al., 2014; Ort and Long, 2014; Moore and Lobell, 2015). Therefore, research to understand the detailed mechanisms causing yield losses under water deficit has received increasing attention, with the aim to improve this trait through breeding (Tester and Langridge, 2010). However, one of the major challenges in the study of water deficit is to determine, non-invasively, when a plant perceives the stress and starts responding to it. This is of importance, as many studies measure yield after water deprivation, which poses the challenge of comparing the difference between growth under normal and water limiting conditions (Tuberosa, 2012; Nelissen et al., 2014). Moreover, yield measurements often represent a destructive quantification at the time of harvest but do not account for the dynamic processes impacting cumulative growth over time. Given that biomass accumulation is largely determined by leaf length increment, understanding the effect of water limitation on leaf growth is a prerequisite to assess the plant response to water deficit. Leaf growth, under non-limiting conditions, has been studied widely and is a dynamic process influenced by developmental, morphological and environmental factors (Reymond et al., 2004; Fournier et al., 2005; Sadok et al., 2007; Walter et al., 2009; Verelst et al., 2013). Given its complexity and the inability to precisely measure growth rates under different severity levels of water deficit, it is challenging to adapt plant growth models to account for environmental stress (Paine et al., 2012).

Monocots encompass most of the agronomically important plant species and are particularly suited to study leaf growth dynamics, due to their temperature-dependent leaf growth and the linear morphology of their growth zone (Bonhomme, 2000; Sadok et al., 2007; Poiré et al., 2010). In monocots, leaf elongation is driven by cell division at the base of the meristem, followed by cell elongation before cells terminally differentiate into mature cells (Durand et al., 1995; Fournier et al., 2005; Sugiyama, 2005; Verelst et al., 2013). It is widely recognized that upon leaf inception, leaf elongation exponentially increases and decays before the final leaf formation (Fournier, 2000; Fournier et al., 2005; Parent et al., 2009; Auzanneau et al., 2011). Both linear and non-linear models have been used to characterize the leaf elongation rate (LER). Linear models assume a steady-state between the exponential and the decay phase and have been used in maize (*Zea mays* L.) and rice (*Oryza sativa* L.) (Sadok et al., 2007; Parent et al., 2009). Non-linear models, which assume an inflection point between the exponential and the decay phase, are typically used to describe whole leaf growth and have been applied to phenotype LER and leaf elongation duration (LED) in perennial ryegrass (*Lolium perenne* L.) (Auzanneau et al., 2011; Voorend et al., 2014). Describing leaf growth in monocots ubiquitously makes use of temperature-dependent growth, described as thermal time (Bonhomme, 2000), but few studies have incorporated further environmental parameters such as the soil water content (Parent and Tardieu, 2014). The effect of the soil water content on LER has been shown to be genetically controlled and is highly heritable (Reymond et al., 2004).

Perennial ryegrass is one of the most widely used forage crops, grown in temperate environments worldwide (Wilkins, 1991; Sampoux et al., 2011). In addition to its economic importance, it is an attractive species for studying leaf growth as it shares many growth features with other monocots and is a perennial species. Moreover, given that biomass is the primary yield target and predominately the product of leaf growth, phenotyping leaf length elongation in perennial ryegrass is of direct agronomic importance. In temperate environments, where perennial ryegrass is cultivated, mild to moderate summer droughts are becoming increasingly frequent (Moore and Lobell, 2015). Although the effect of severe drought has been the subject of many studies, terminal responses to near lethal stress differ from observations under mild or moderate stress which already limit growth (Claeys and Inzé, 2013; Verelst et al., 2013). Water stress is a dynamic process. Therefore, the plant response to particular stress phases could be used as a diagnostic tool for improving drought tolerance (Tardieu, 2013). By determining at which point a plant limits and arrests growth, cultivars or genotypes being able to cope with short term water limitation could be selected and used to improve biomass production under future climate conditions.

In this work, we sought to resolve the fundamental question when perennial ryegrass plants start to limit or terminate leaf growth in response to stress caused by water limitation, based on the hypothesis that a reduction in leaf growth can be used as an indicator of a stress response. Specifically, we aimed at (i) establishing a standardized and repeatable experimental setup to elicit water deficit stress in perennial ryegrass, (ii) profiling LER at high temporal frequency in a non-invasive manner, (iii) calculating thermal growth rates of perennial ryegrass genotypes independent of temporal developments and (iv) identifying critical thresholds when plants slow and halt growth in response to water deprivation.

## Materials and Methods

### Plant Material and Growth Conditions

Two genotypes of the perennial ryegrass variety “Arara,” designated “Arara A” and “Arara B,” were used throughout the experiments. Both genotypes were vegetatively propagated into clonal replicates, each consisting of 20 tillers. Plants were grown in plastic pots (ø15 and 12 cm height) filled with 450 g of commercial potting mix substrate (‘Spezialmischung 209,’ RICOTER Erdaufbereitung AG, Aarberg, Switzerland) under regular irrigation and fertilization in a greenhouse. Four to six weeks after clonal propagation, plants were transferred into a climate chamber (Conviron, Winnipeg, Canada) under controlled conditions with a light/dark photoperiod of 16/8 h and a light intensity of 275 μmol photosynthetically active radiation (PAR) m^-2^ s^-1^. The climate chamber was equipped with a 2:1 mixture of fluorescent lamps of two types (T5 FQ 54W/840 HO, Osram GmbH, Munich, Germany and T5 FH054W/GRO G5 F 54W, Havells Sylvania Europe, Ltd., London, United Kingdom). The day/night temperature was 25/15°C, and relative air humidity was set to 50% ( ± 20%).

In order to test for the genetic variability of water deficit response traits, five perennial ryegrass genotypes, designated 1299 (var. “Vigor,” forage type), 3052 (ecotype from Lithuania), 3891 (ecotype from Ukraine), 3901 (ecotype from Ukraine) and genotype 3964 (var. “Recolta,” turf-forage type) were used. Plants were vegetatively propagated into four replicates consisting of around 20 tillers each and grown as described above.

### LER Measurements

**FIGURE 1:**
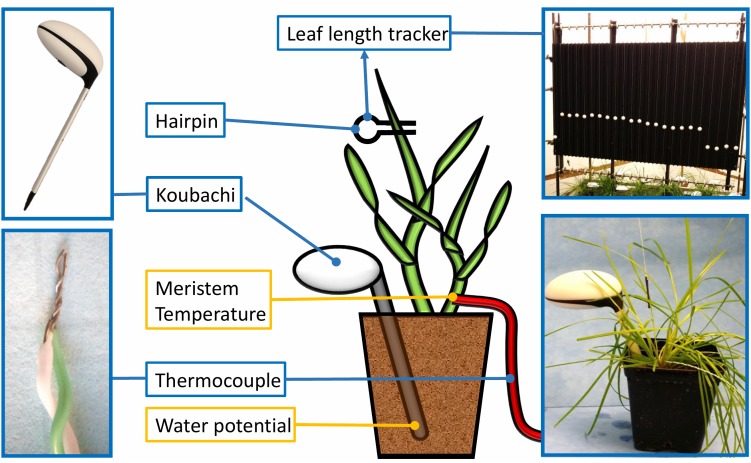
Phenotyping platform for chronological profiling of the leaf elongation rate (LER), the soil water potential and micrometeorological variables. The figure illustrates a perennial ryegrass plant with the following sensors connected: a wireless Koubachi plant sensor used to measure the soil water potential, the Leaf Length Tracker (LLT) used to measure leaf growth over time and a thermocouple used to record the temperature in the meristem zone.

Leaf growth measurements were made using the methods described by Nagelmüller et al. (2016). The leaf tip of the youngest growing leaf of a representative tiller (with at least three leaves formed) was attached with a hair pin to a string and kept taut over a reverse roller using weights of 20 g. White plastic beads (ø20 mm, 7 g) were threaded onto the strings and placed on the growth array to provide artificial landmarks for image-based marker tracking that allowed registration of leaf increment in 2 min intervals (Figure 1). Images of the growth array were taken with a LupusNET HD camera with 2.1 pixel mm^-1^ resolution (LUPUS-Electronics^®^ Gmbh, Landau, Germany) installed at an approximate distance of 1.5 m. Image sequences of each experiment were analyzed with the LLT software (Nagelmüller et al., 2016).

### Water Deprivation

Soil moisture sensors integrated in a wireless microclimate sensing system (WiFi Plant sensor, Koubachi, Switzerland) measured the soil matric potential using the energy needed to change the temperature of a ceramic plate with defined porosity (Liebisch et al., 2013). Each sensor was calibrated individually and data collected every 4 h at a depth of 7 cm. Meristem temperatures of six plants per experiment were measured with a K type thermocouple (GTF 300, Greisinger, Germany; 0.2 mm diameter) inserted into the tiller at meristem height (Figure 1). All temperatures referred to hereafter are meristem temperatures.

To establish a standardized experimental setup eliciting a wide range of stress induced by water deprivation in perennial ryegrass, seven clonal replicates of “Arara B” were selected. Throughout the experiment, “Arara A” was kept as a control with 15 clonal replicates under well-watered conditions (experiment E1). To induce water deprivation stress, plants were transferred 4 to 6 weeks after clonal propagation in a controlled environment of a growth cabinet. After an initial adaption phase of 1 week under well-watered conditions, the experimental plants (“Arara B”) were subjected to water deprivation for 130 h and re-watered after and grown for 35 h, while the control plants (“Arara A”) were kept under regular water management regimes. The water deprivation experiment was repeated twice using the same climate settings, with 10 clonal replicates for each experiment (experiments E2 and E3).

To quantify how the increasing soil moisture deficit translated into physiological stress perceived by the plants, the relative water content (RWC) of the experimental and the control plants was measured. At least two mature leaves per plant were cut using a sharp blade and stored in a zip-lock bag on ice before weighing for fresh weight (FW). To measure turgid weight (TW), the samples were then re-hydrated for 8 h in a water-filled petri-dish. Dry weight (DW) was then measured after drying the samples for a minimum of 24 h at 80°C. RWC was calculated using the formula described by Smart and Bingham (1974), Eq. 1).

(1) $$\mathrm{RWC}=\frac{\mathrm{FW}-\mathrm{DW}}{\mathrm{TW}-\mathrm{DW}}\times 100$$

### Statistical Analysis

The statistical analysis was implemented in the open source R statistical environment (version 3.1.0; R Development Core Team, 2005). LER data were summarized into hourly time intervals from the start of experiment. Environmental data from the climate cabinet and meristem temperature (T) were summarized using the mean, within an hourly time frame. LER was determined using the difference between the maximum (L_1_) and minimum (L_0_) leaf length (mm), divided by the difference in time (minutes) between the two measurements (t_1_–t_0_). This was then multiplied by 60 to give the hourly LER rate (mm h^-1^) as shown in Eq. 2. Soil moisture (hPa) data was log_10_ transformed and then hourly data were imputed by using a Loess fit (‘loess’ function in R, Cleveland et al., 1992). Only data before re-watering were used for the Loess fit, otherwise this led to a severe underestimation of the final soil moisture.

(2) $$\mathrm{LER}=\frac{{\mathrm{(L}}_{1}-L_{0})}{\left(t_{1}-t_{0}\right)}\times 60$$

The summarized data were then used as input for the Tri-phase function. Briefly, data from the first 24 h were used for a linear model to calculate *a*, the relationship between LER and T with an intercept fitted through 0, as shown in Eq. 3. Once *a* was determined, the relative growth rate (RGRa) was calculated by division of LER by *aT* across all time points (Eq. 4).

(3) LER=0+aT

(4) $$\mathrm{RGRa}=\frac{\mathrm{LER}}{aT}$$

Average RGRa was estimated per quarterly Ψ log_10_ (hPa) (i.e., 2.00–2.25, 2.25–2.50, 2.50–2.75, etc.) and then considered those above 0.9 RGRa to be “normal” and those below 0.2 RGRa to have “arrested.” In case the mean RGRa dropped below 0.9 and returned above 0.9 in the next increment, RGRa was still considered “normal.” Additionally, the maximum quarter Ψ log_10_ (hPa) > 0.9 RGRa is retained in the subset data to improve the estimate of Σ. From these data, we then estimated c (Eq. 5) and defined Σ and σ to be when c intercepts RGRa at 1 and 0, respectively.

(5) $$\frac{\mathrm{LER}}{aT}=i+c\Psi$$

## Results

### Induction of Water Deficit Stress in Perennial Ryegrass

To induce water deficit stress in perennial ryegrass, the experimental plants were subjected to water deprivation for 130 h and re-watered after, while the control plants were kept under regular water management regimes. Over the course of 130 h, the soil water potential Ψ (hPa) increased steadily in the pots of the plants without watering. For the first 24 h, the average Ψ was below 2 log_10_ (hPa), for the second 24 h period below 3 log_10_ (hPa) and reached a plateau after 96 h at around 4.3 log_10_ (hPa) (data not shown).

**FIGURE 2:**
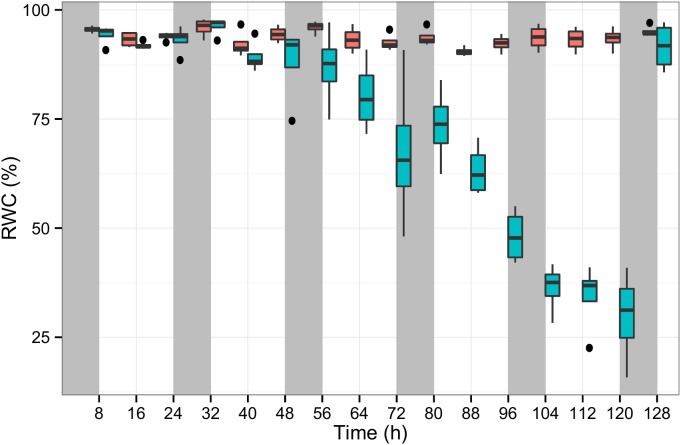
The relative water content (RWC) of perennial ryegrass plants measured in at least two leaf tissue samples per plant. The blue boxplots show the RWC values of seven experimental plants subjected to water deprivation for 130 h and after re-watering. The red boxplots show 15 well-watered control plants along the same experimental duration (x-axis). Night periods are indicated with gray shading.

To quantify the physiological stress perceived by the plants, the RWC was measured in the leaves of control and stressed plants across 130 h. In well-watered plants, RWC was on average 94% during the stress experiment, while in the plants subjected to water deprivation over the first 56 h RWC was on average 92% and dropped significantly to 60, 45 and 27% after 72, 96, and 120 h, respectively. Eight hours after re-watering RWC had returned to 92% (Figure 2).

### Leaf Growth Measurement Under Water Deficit Stress

**FIGURE 3:**
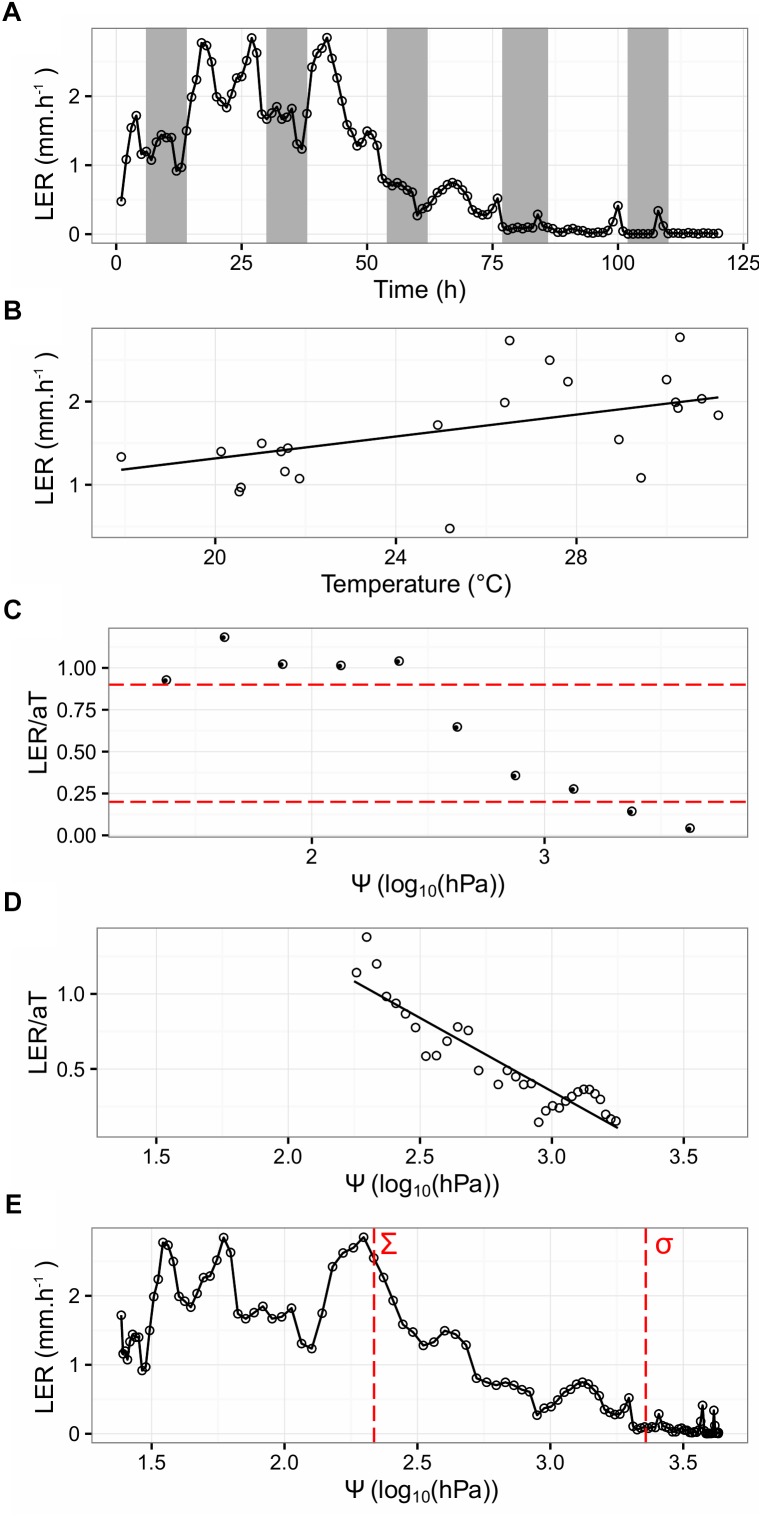
Parameter determination of the Tri-Phase function, illustrated on a single tiller from one clonal replicate of the “Arara B” genotype. **(A)** Shows the LER, summarized hourly over a period of 120 h (hollow circles). Night periods are indicated with gray shading. **(B)** Shows LER, plotted against the meristem temperature (T) from the first 24 h of the experiment (hollow circles). The fitted regression (a) is given as a black solid line and intercepts at 0. **(C)** Shows the normalized growth rate (LER/aT) per quarterly soil moisture (Ψ) interval (circles). The red dashed lines represent cutoff values of 0.9 and 0.2 LER/aT. **(D)** Shows hourly LER, plotted against Ψ from the filtered data (0.2 < LER/aT < 0.9). The linear regression of the hollow circles is given as a solid black line. **(E)** Shows LER, plotted against Ψ with estimated Σ and σ, demarcating where LER decreases and stops, shown as vertical red dashed lines at 2.4 and 3.4 log_10_(hPa), respectively.

The Leaf Length Tracker (LLT) was used to measure leaf growth in response to increasing water deficit and diurnally fluctuating temperatures (Nagelmüller et al., 2016). To precisely record the temperature in the growing zone, a thermocouple was inserted into the meristem. Data from all the sensors, in combination with the image sequences analyzed with the LLT software, allowed chronological profiling of LER, in response to Ψ and micrometeorological variables (Figure 3).

For one tiller of each of the seven “Arara B” clonal replicates, LER (measured in mm per hour) was recorded over time under water limiting conditions (as exemplified for one selected tiller in Figure 3A). Generally, LER in perennial ryegrass was higher during daytime and a good correlation was found between LER and the meristem temperature (*R^2^* = 0.92, *P* < 0.05). Beyond the diurnal fluctuations, LER significantly dropped after 48 h of water deprivation, reaching 0 after 76 h. In order to confirm that water deprivation was the cause of leaf growth arrest, the plants were re-watered after 130 h of stress treatment for continued phenotyping of an additional 35 h. In all cases, leaf growth sharply resumed after watering (Supplementary Figure S1).

In order to account for the temperature (T) and its influence on growth, LER was plotted against T from the first 24 h of the experiment, under the assumption that up to this time point, LER was not affected by Ψ and LER intercepts T at 0°C, as shown in Eq. 2.

As indicated by the fitted regression, given as a black solid line in Figure 3B for the same selected tiller as above, LER augmented with increasing T by the slope *a*, which amounted to an average of 52.9 μm°Ch^-1^ over all the seven tillers.

To illustrate thermally corrected LER in response to increasing water deficit, the ratio between observed LER and expected *a*T (LER/*a*T) per quarterly Ψ interval was calculated (as exemplified for the same selected tiller in Figure 3C). For the interval 0.2 ≤ LER/*a*T ≤ 0.9, hourly LER (thermally corrected) was plotted against Ψ and used to estimate *c*, for accurate quantification of the relationship between thermally corrected LER and Ψ. This was done using a linear model, as shown in Eq. 5 with an intercept *i*.

The slope of the solid black line in Figure 3D determined *c* and indicated a strong correlation (*R^2^* = 0.84, *P* < 0.05) between LER and Ψ. Eliminating data not being directly affected by Ψ (i.e., data outside the interval 0.2 ≤ LER/*a*T ≤ 0.9) improved the estimation of the genotypic response *c* to Ψ (data not shown). Finally, upper sigma (Σ) and lower sigma (σ) were defined as *c* intercepts LER/*a*T at 1 and 0, respectively, and demarcate where LER in response to Ψ decreases and stops (Figure 3E). On average over the seven “Arara B” clonal replicates, Σ and σ were estimated to 2.70 and 3.83 log_10_ (hPa), respectively.

### Definition of the Tri-Phase Function

**FIGURE 4:**
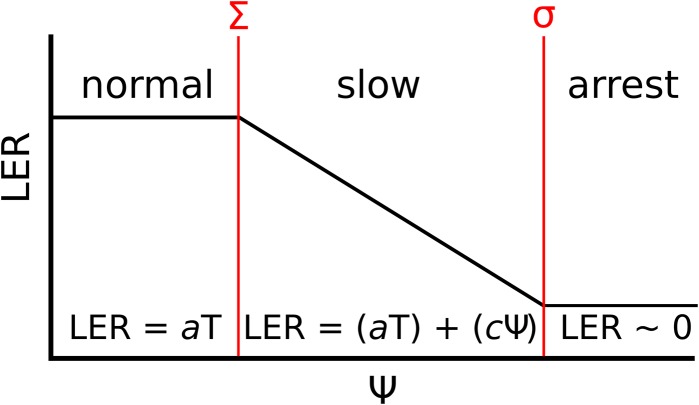
The Tri-Phase function describing the LER under water deficit. The diagram shows three phases of LER (y-axis); “normal,” “slow,” and “arrest,” in response to increasing soil water potential (x-axis, Ψ). The three phases are demarcated by two points (given in red), when growth begins to slow (Σ) and when growth arrests (σ). Under normal growth (Ψ < Σ), LER is attributable to the meristem temperature (T) and the genotypic response to it (*a*). When Σ ≤ Ψ ≤ σ, LER is dependent upon *a*T and the genotypic response to Ψ (*c*). In the final arrest phase (Ψ > σ), LER equals to 0 and is therefore not dependent upon *a* or *c*.

Based on our results, leaf growth under water deficit in perennial ryegrass can best be described by three phases, referred to as a “normal,” a “slow” and an “arrest” phase. These phases are demarcated by the two Ψ values Σ and σ, as LER decreases and ceases, respectively (Figure 4). The first “normal” phase corresponds to well-watered conditions (Ψ < Σ), when LER is dependent upon T and the genotypic response to it (*a*) (Bonhomme, 2000; Sadok et al., 2007). In the second “slow” phase (Σ < Ψ > σ), LER is dependent upon the genotypic response to temperature (*a*T), the soil water potential (Ψ) and the genotypic response to it (*c*) (Reymond et al., 2004). In the last “arrest” phase (Ψ > σ), leaf growth has effectively stopped (LER ≈ 0) and is therefore not subject to other environmental factors than Ψ. In order to efficiently estimate the parameters *a, c*, Σ and σ, a script (referred to as the Tri-Phase function) in R language (R Development Core Team, 2005) was developed and allowed highly automated proceeding of the raw data revealed by the phenotyping platform.

### Accuracy and Applicability of the Phenotyping Platform and the Tri-Phase Function

**FIGURE 5:**
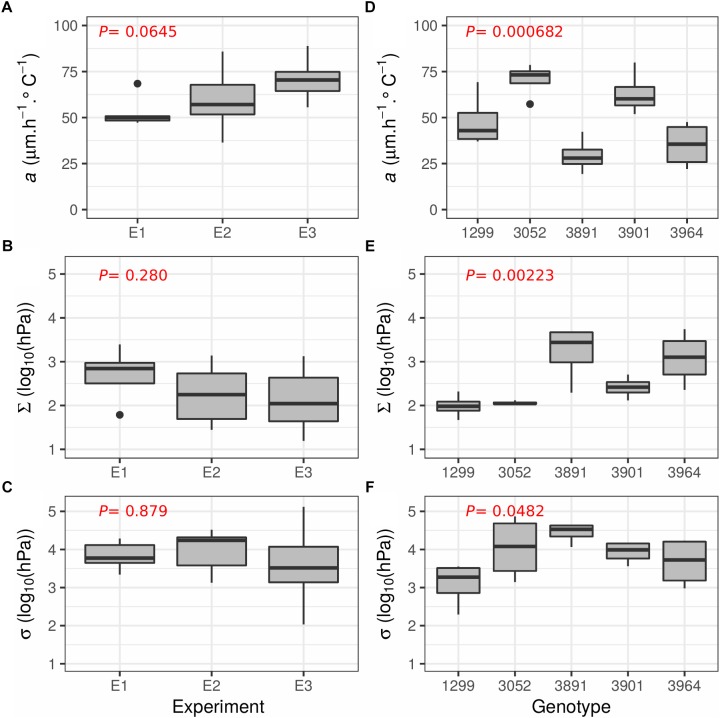
Boxplots illustrating the reproducibility of the phenotyping method and the Tri-Phase function across different experiments and genotypic responses. **(A–C)** Shows the results from the three replicated experiments (E1, E2, and E3) using the genotype “Arara B” with 7, 10, and 10 replicates per experiment, respectively. **(D–F)** Shows the response of the five different perennial ryegrass genotypes 1299, 3052, 3891, 3901 and 3964, each evaluated with four replicates. **(A,D)** Shows the LER per degree (*a*, μm h^-1∘^C^-1^), **(B,E)** the estimates when leaf elongation slows [Σ, log_10_ (hPa)], and **(C,F)** when leaf growth arrests [σ, log_10_ (hPa)]. *P*-values from a one way ANOVA are given in red at the top left of each graph.

To test the accuracy and reproducibility of the phenotyping platform and the Tri-Phase function to estimate *a, c*, Σ and σ, the experiment described above (hereafter referred to as E1) was repeated in two consecutive follow-up experiments (E2, E3, Figure 5). For both E2 and E3, 10 clonal replicates of the “Arara B” genotype were analyzed. The results of the Tri-Phase function for *a*, Σ, and σ are shown in Figure 5A–C, respectively. Across the three experiments E1 to E3, the mean values for *a*, Σ and σ was estimated to 62.5 μm°Ch^-1^, 2.30 log_10_ (hPa) and 3.90 log_10_ (hPa), respectively. Using ANOVA, no significant (*P* > 0.05) differences were found between the replicated experiments, demonstrating the reproducibility of the measurements.

Finally, five different perennial ryegrass genotypes were used to assess the utility of the phenotyping platform and the Tri-phase function to uncover genotypic differences. Each of the five genotypes, selected from an association mapping panel established to study abiotic stress and plant architecture (Jonavičienė et al., 2014; Aleliūnas et al., 2015; Statkevičiūtė et al., 2015), was represented with four clonal replicates. The results of *a*, σ, and Σ are shown in Figure 5D–F, respectively. When assessing the different genotypes by ANOVA, significant differences were found for all parameters (*P* < 0.05). The genotype 3891 showed the lowest genotypic leaf thermal growth rate (*a* = 29.4 μm°Ch^-1^) but the best tolerance to water deficit [Σ = 3.22 and σ = 4.44 log_10_ (hPa)]. Genotype 1299 showed a modest *a* (48.0 μm°Ch^-1^) but was highly sensitive to water shortage [Σ = 1.99 and σ = 3.10 log_10_ (hPa)]. The two genotypes with the fastest growth rate were 3052 and 3901 with a mean *a* value of 70.6 and 63.0 μm°Ch^-1^, respectively. Both decreased growth relatively early [Σ = 2.06 and 2.41 log_10_ (hPa), respectively] but stopped growth comparatively late [σ = 4.04 and 3.93 log_10_ (hPa)]. The genotype 3964 showed slow growth (*a* = 35.0 μm°Ch^-1^) but performed moderately under water limiting conditions [Σ = 3.08 and σ = 3.67 log_10_ (hPa)].

## Discussion

We have established a non-destructive and largely automated phenotyping platform to collect graminoid leaf growth data at high temporal resolution, applied it to an experimental setup that reliably elicited a wide range of water deficit stress to perennial ryegrass genotypes and were able to precisely quantify the genotypic response of water deficit on leaf growth. According to our results, leaf growth in response to water deficit is not linear but can be described by three phases, demarcated by the growth reduction point (Σ) and the growth arrest point (σ). The first phase is determined by temperature-dependent growth, followed by a growth reduction proportional to soil moisture availability and an arrest phase where leaf growth halts. The results of the phenotyping platform and the Tri-Phase function are highly reproducible and uncovered genotypic differences in a small but diverse set of perennial ryegrass genotypes. Importantly, the Tri-Phase function tackles a significant challenge when studying the effect of water limitation on plants, since it identifies when a plant responds to water stress. To the best of our knowledge, no existing function or model can accurately pinpoint when a plant restricts growth due to water stress.

Key to the Tri-Phase function’s success is that it takes into account environmental parameters such as temperature and soil moisture to describe plant growth. In many studies, even with replicated experiments, environmental differences are often overlooked and can result in a strong genotype by environment interaction which hampers a precise genetic dissection of the drought response (Coupel-Ledru et al., 2014; Kumar et al., 2014). However, as demonstrated by Reymond et al., 2004, modeling based on time independent environmental factors provides a robust means to quantify genotypic responses to adverse conditions. In temperate regions, short and non-lethal drought spells may still limit growth without visible stress phenotypes (Skirycz et al., 2010, 2011a,b; Baerenfaller et al., 2012), thereby affecting biomass yield. From an agronomic perspective, leaf elongation is a key trait for biomass accumulation, which is of high importance for forage crops, as vegetative material forms yield. Therefore, quantification of the genotypic response to water limitation, in the context of premature growth, is a viable trait for yield improvement in forage crops. For these reasons, we propose to determine and improve Σ in breeding programs to produce elite cultivars with improved yield under future climatic conditions of temperate environments.

The phenomenon of temperature-dependent leaf growth has been well-documented in many grass species including perennial ryegrass, tall fescue (*Festuca arundinacea* Schreb.), wheat (*Triticum aestivum* L.), barley (*Hordeum vulgare* L.) and maize (Durand et al., 1995; Reymond et al., 2004; Laidlow, 2009; Auzanneau et al., 2011; Nagelmüller et al., 2016). In the Tri-Phase function, temperature-dependence was taken into account to set a basal temperature independent growth rate by the calculation of *a* from the first 24 h. Moreover, the thermally corrected leaf extension suggested a steady-state leaf growth, as reported in wheat, maize, rice, and tall fescue (Fournier et al., 2005; Sadok et al., 2007; Parent et al., 2009; Nagelmüller et al., 2016). In contrast, Auzanneau et al. (2011) and Voorend et al. (2014) used a Beta-sigmoid function to describe leaf growth in perennial ryegrass and did not observe a steady-state. This discrepancy was further investigated by applying the above described experimental setup to 15 clonal replicates of the “Arara A” genotype under well-watered conditions over 130 h (Supplementary Figure S2). Although a slow decrease of the temperature-independent growth rate (LER/*a*T) was detected, the reduction rate is negligible in our experimental setup and did not affect the estimation of growth slow (Σ) and growth arrest points (σ).

Many complex physiological processes can be defined by distinct chronological phases that dissect the underlying molecular mechanisms into meaningful stages. For example, the life cycle of many plants, such as soybean (*Glycine max* L.) and Arabidopsis (*Arabidopsis thaliana* L.), can be described by well-characterized growth ratings (Fehr and Caviness, 1977; Boyes et al., 2001). Similarly, the progression of biotic stress and pathogen infection can in many cases be categorized by phenotypic observation (Windram et al., 2012). Such structuring allows particular stages of a complex process to be targeted for further analysis, which ultimately can lead to improvement of growth or disease resistance. To our knowledge, such profiling under water deficit stress remains elusive, especially with respect to *in situ* profiling. It is well-known that abscisic acid (Raghavendra et al., 2010) and osmolytes such as proline accumulate in response to water deprivation (Delauney and Verma, 1993). Similarly, leaf water content decreases (Smart and Bingham, 1974; Jonavičienė et al., 2012) and stomata close (Delauney and Verma, 1993). But how these metabolites and processes relate to growth and growth changes is largely unknown. Therefore, providing tools to describe leaf growth in response to water limitation will enable researchers to target and unravel the mechanisms behind specific processes.

The possibility to identify genotypic differences in response to water deficit opens a number of opportunities: for example, the determination of Σ, σ, *a* and *c* in genetically characterized populations would allow association mapping and the identification of QTL underpinning interesting phenotypes (Kearsey and Pooni, 1998). Such a strategy would be of value for trait introgression into elite germplasm by marker assisted selection (MAS) (Peleman and van der Voort, 2003), which has, for example, improved drought tolerance in rice breeding programs (Kumar et al., 2014). Additionally, the phenotyping platform and the Tri-Phase function could also be combined with molecular techniques to study transcriptome responses, either at a single gene level (Livak and Schmittgen, 2001) or genome-wide (Mortazavi et al., 2008). Such data could be used for the reconstruction of gene regulatory networks (Penfold and Wild, 2011; Windram et al., 2012). Gene regulatory networks enable uncovering genes and corresponding pathways that are regulating growth, by chronological profiling of genes which modulate expression at key physiological changes (Σ and σ).

In this study, the LLT method (Nagelmüller et al., 2016) was used in a laboratory setup with few modifications. Further optimisation of the setup might help to increase precision and throughput of the system in the future. For example, the sensors can be substituted with alternative equipment such as rotational displacement transducers to monitor LER and gravimetric approaches to measure soil moisture, as described by Reymond et al. (2004) or Laidlow (2009). Moreover, the LLT setup can be adapted to the field. Nagelmüller et al. (2016) have already shown the potential for LER tracking under extreme conditions and instead of Koubachi sensors, soil moisture can be monitored using a number of sensors and devices, such as tensiometers or lysimeters (Titus and Mahendrappa, 1996).

Beyond the characterisation of leaf growth in perennial ryegrass under water deficit, the versatility of the method described here allows its application to other grass and woody species with largely linear growth features, or the study of other abiotic stresses such as salinity, where in place of decreasing water availability, the soil might be subject to increasing salt concentrations. Given that a dosing treatment is possible, osmotic stress (induced by polyethylene glycol or mannitol), heavy metal toxicity (aluminim or cadmium) or chemical agents [such as fertilizers or pesticides (Claeys et al., 2014)] can also be monitored. Thus, the tools presented here are adaptable to a wide range of scenarios, to study plant growth in response to adverse conditions and to improve stress tolerance in monocot crop species by breeding.

## Conclusion

Two major challenges persist in quantifying the response of water deficit stress in plants; the effect of environmental variables and the precise identification of growth reduction as a consequence of water deficit. The Tri-Phase function described here takes into account these factors and quantifies a reproducible genotypic response to water deficit, offering new opportunities to study and improve this trait by breeding. Firstly, the precision in identifying at which point a plant reduces and arrests growth due to water deficit allows the mechanisms underlying these responses to be investigated. Secondly, the quantification of these parameters in populations will allow for targeted selection of superior genotypes in elite germplasm or for QTL identification and MAS strategies. Moreover, the experimental setup can be adapted to other growth limiting factors or for field evaluations, to study growth parameters in Poaceae species, including the economically most important cereal, forage and energy crops. We have demonstrated the use of the phenotyping platform and Tri-Phase function in perennial ryegrass where leaf biomass is the major yield component. Given the intrinsic link between leaf growth and biomass accumulation, the Tri-Phase function can be used in perennial ryegrass to curtail yield depression due to mild or moderate summer droughts in temperate environments. The work presented here has strong application for both fundamental and applied research to improve crop productivity.

## Data Availability

The Tri-Phase function with the examples are available at https://github.com/stevenandrewyates/TriPhaseFunction.

## Author Contributions

AW, GB, and BS conceived the study. SY and KJ conducted the experiments and performed data analysis and interpretation. SN, FL, RK, and NK assisted in the experimental setup and data analysis. SY and KJ drafted the manuscript, which was improved by AW, GB, RK, and BS. All authors read and approved the final manuscript.

## Conflict of Interest Statement

The authors declare that the research was conducted in the absence of any commercial or financial relationships that could be construed as a potential conflict of interest.

## Supplementary Material

FIGURE S1 Extension of perennial ryegrass leaves under water deficit conditions. The graph shows leaf length extension (y-axis, mm) of seven leaves using clonal replicates of a single genotype. The x-axis shows the time (h) since the start of the experiment. From time 0 onwards, water was withheld until the re-watering after five days (black line). The growth of individual tillers is shown as colored lines.

FIGURE S2 Relative growth rates of single tillers from 15 clonally replicated plants of the perennial ryegrass genotype Arara A under well-watered conditions. The x-axis shows time (h). The y-axis illustrates the observed leaf elongation rate (LER) divided by the expected growth rate per temperature (*a*T). Results are given per hour, shown as a continuous black line. The linear fits of LER/*a*T versus time are indicated by the blue line. The averaged data of the 15 tillers indicated that LER/*a*T decreased at a rate of 0.005 (± 0.002) per hour.
