# Supplementary figures and images for: Phenotyping a Dynamic Trait: Leaf Growth of Perennial Ryegrass Under Water Limiting Conditions
Source: Front Plant Sci. 2019 Mar 22;10:344. doi: 10.3389/fpls.2019.00344 (PMC6440318; doi:10.3389/fpls.2019.00344)

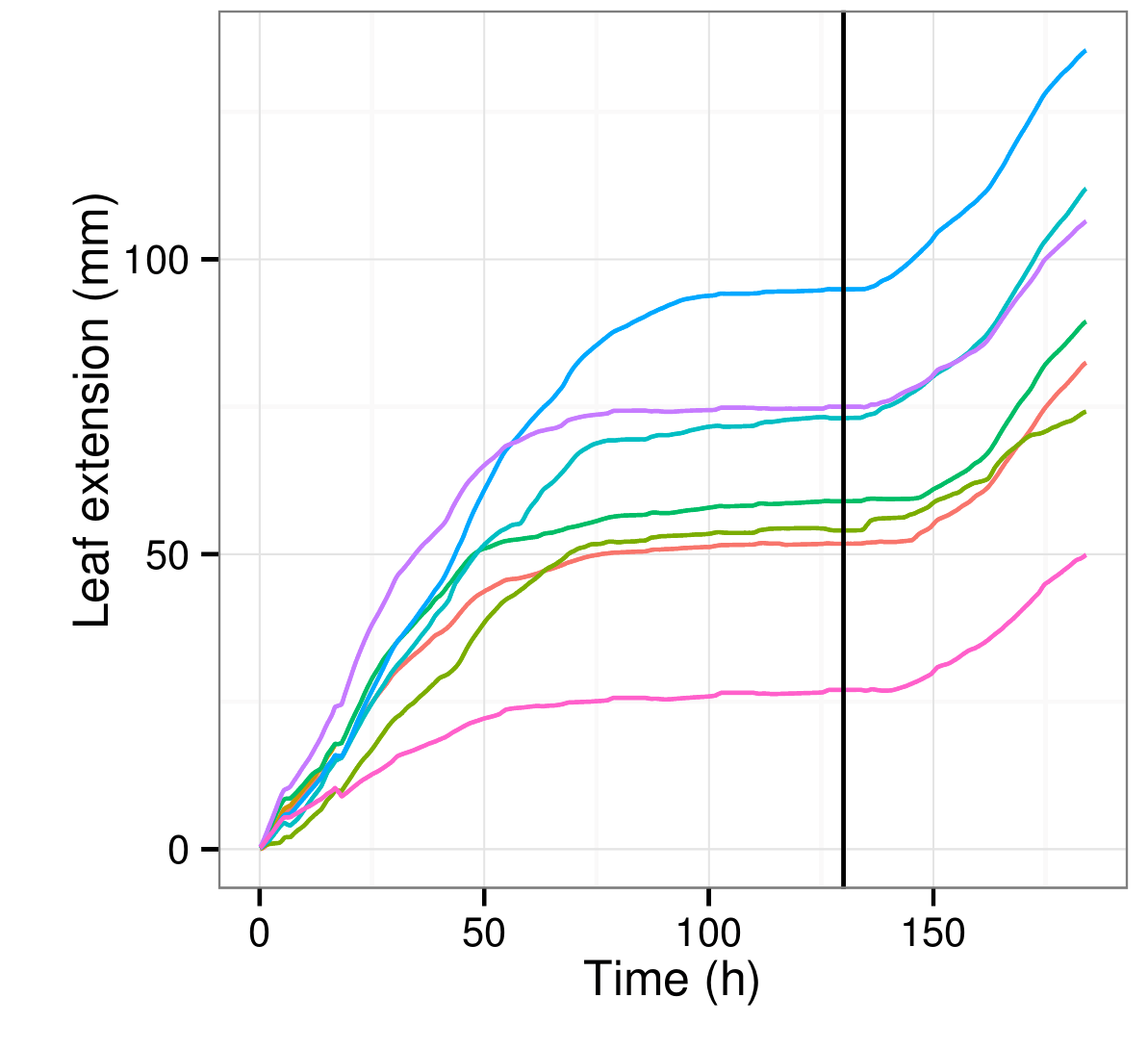

Supplement: FIGURE S1 — Extension of perennial ryegrass leaves under water deficit conditions. The graph shows leaf length extension (y-axis, mm) of seven leaves using clonal replicates of a single genotype. The x-axis shows the time (h) since the start of the experiment. From time 0 onwards, water was withheld until the re-watering after five days (black line). The growth of individual tillers is shown as colored lines. [file Image_1.TIF]

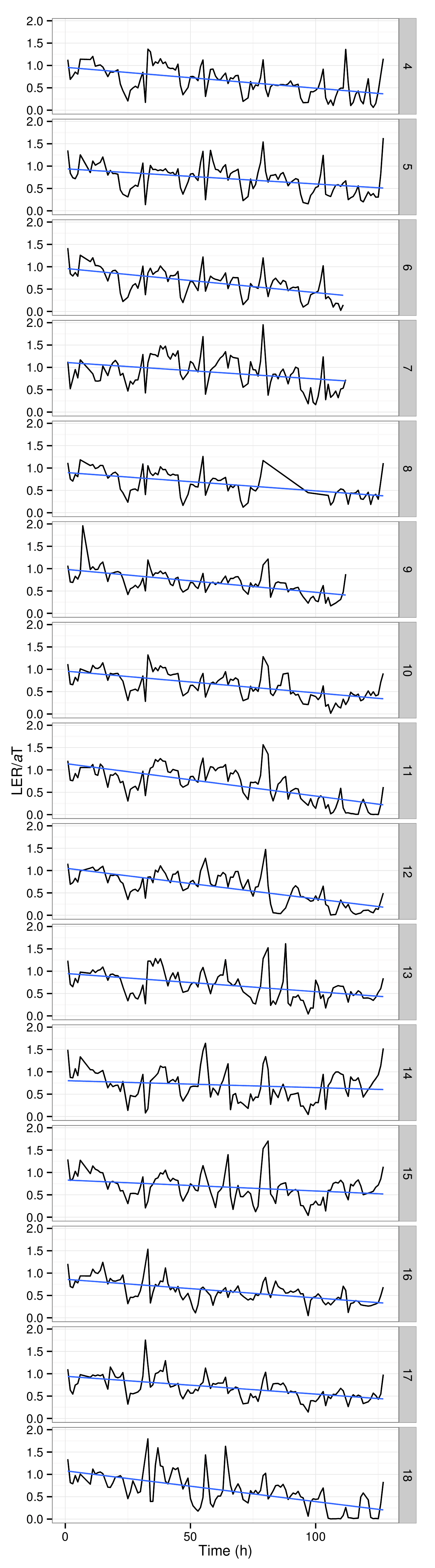

Supplement: FIGURE S2 — Relative growth rates of single tillers from 15 clonally replicated plants of the perennial ryegrass genotype Arara A under well-watered conditions. The x-axis shows time (h). The y-axis illustrates the observed leaf elongation rate (LER) divided by the expected growth rate per temperature (aT). Results are given per hour, shown as a continuous black line. The linear fits of LER/aT versus time are indicated by the blue line. The averaged data of the 15 tillers indicated that LER/aT decreased at a rate of 0.005 (± 0.002) per hour. [file Image_2.TIF]
